# Application of DNA Methylation–Based Age Estimation to Construct an Age Structure of Humpback Whales in a Newly Emerged Wintering Ground Around Hachijojima Island, Tokyo Metropolis, Japan

**DOI:** 10.1002/ece3.70854

**Published:** 2025-02-10

**Authors:** Kohei Igarashi, Atsushi Tanabe, Hiroeki Sahara, Reiko Nozaki, Hidehiro Kondo, Taiki Katsumata, Shingo Tamura, Tadashi Yamakoshi, Mizuki Mori, Marin Miyagi, Gen Nakamura, Naohisa Kanda, Hiroto Murase

**Affiliations:** ^1^ Laboratory of Cetacean Biology Tokyo University of Marine Science and Technology Tokyo Japan; ^2^ Laboratory of Highly‐Advanced Veterinary Medical Technology Azabu University School of Veterinary Medicine Sagamihara Kanagawa Japan; ^3^ Laboratory of Biology Azabu University School of Veterinary Medicine Sagamihara Kanagawa Japan; ^4^ Laboratory of Genome Science Tokyo University of Marine Science and Technology Tokyo Japan; ^5^ Institute of Cetacean Research Tokyo Japan; ^6^ Hachijojima Island Tourism Association Tokyo Japan; ^7^ Hachijo Town Office Tokyo Japan

**Keywords:** Asia, cetacean, DNA methylation (DNAm), epigenetics, humpback whale, Japan, marine mammal, North Pacific

## Abstract

Using a noninvasive DNA methylation (DNAm)–based age estimation method, we investigate the age structure of humpback whales that newly emerged around Hachijojima Island, Tokyo Metropolis, Japan to uncover the role of this area for this species. We measured DNAm frequencies at three age‐related genes (*GRIA2*, *CDKN2A,* and *TET2*) from 26 biopsy skin samples of 21 unique humpback whales (15 males and 6 females) randomly collected in the winters of 2018–2021 and estimated their ages using the known age estimation model for humpback whales. The estimated ages of the 21 individuals were 2.95–30.40 years old with a mean of 12.02, and the resulting age structure in 5‐year increments was roughly normally distributed with a peak at 10.00–14.99 class, suggesting the dominance of young adult males in this water. The observations (young males, male aggressive behavior for mating, whale song, and mother–calf pair) indicated that newly emerged humpback whales appeared to utilize Hachijojima Island as their new wintering ground, expanding the northern limit of the wintering area in the western North Pacific from previously known.

## Graphical abstract

The age structure of humpback whales that newly emerged around Hachijojima Island, Tokyo Metropolis, Japan, was investigated using a noninvasive DNA methylation (DNAm)–based age estimation method. The resulting age structure in 5‐year increments was roughly normally distributed with a peak at 10.00–14.99 class, suggesting the dominance of young adult males in this water. The observations indicated that newly emerged humpback whales appeared to utilize Hachijojima Island as their new wintering ground, expanding the northern limit of the wintering area in the western North Pacific from previously known.

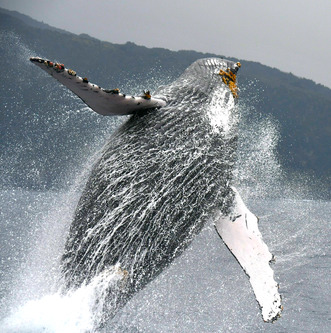

## 1 Introduction

Describing the age structure of individuals in a population leads us to implement successful and effective wildlife conservation and management. Without the age structure data, it is not easy to understand the population's biological and ecological characteristics that influence its long‐term persistence, such as the type of social structure they form and the differences in habitat use depending on their life history. Additionally, age is essential information to evaluate the population's current status and predict its future status.

The age of each individual (chronological age measured by time) can be easily determined if the individual can be tracked from birth through life. Unfortunately, tracking long‐lived, highly migratory large animals such as whales is difficult because it is time and resource intensive. Therefore, their ages have been estimated indirectly by counting annual growth layer groups (GLGs, a group of layers occurring with cyclical and predictable repetition) observed in bones, teeth, baleen plates, and earplugs. Presently, teeth for toothed whales and earplugs for baleen whales are widely used (Hohn 2018). One major drawback of the age assessment method using such age traits is that it requires lethal sampling in many cases. From a conservation biology perspective, lethal sampling should be avoided for research on protected species, and it is also not appropriate for continuous surveys of living organisms (Mayne, Berry, and Jarman 2021; Polanowski et al. 2014). Additionally, counting GLGs of the age traits is technically challenging to master, and often involves a potential subjective error among age readers during visual measurements (Kitakado, Lockyer, and Punt 2013), and is very time‐consuming. Recently, another type of age estimation method has been developed using a relatively new age trait, racemization, which occurs in the eye lens when the L‐form of aspartic acid changes to D‐form with age (Boye et al. 2020). However, eye samples can also be obtained lethally. Regarding nonlethality, the photo identification method can track and age individual whales without any sacrifice because it focuses on differences between individuals in external morphology (natural markings), such as body coloration and fin shape. This method, however, is practically challenging for long‐lived wild whales because it needs to know their age to start from in most cases, and it takes a long time to develop a sufficient amount of the data set. Therefore, the development of new methods has been thought necessary to overcome these limitations.

One candidate for nonlethal age estimation utilizes the correlation between biological age determined by DNA methylation (DNAm) frequency as an index and chronological age that is directly or indirectly known prior to the analysis. DNAm is an epigenetic modification that involves the covalent bond of a methyl group to cytosine at a CpG (cytosine–phosphate–guanine) sites in a DNA sequence (Horvath 2013). A correlation existed between the DNAm frequency (also called as biological or epigenetic age) at CpG sites within some specific genes and the chronological ages of the individuals in humans and mice (Maegawa et al. 2010; Hannum et al. 2013; Horvath 2013) and was used to develop age estimation models. Because DNAm frequency can be measured using DNA from a tissue sample, we only need a tiny amount of tissue from each whale collected in a nonlethal, minimally invasive way. The development of DNAm‐based age estimation models has been increasing in cetaceans because the accuracy of the model is comparable to the conventional methods, and the technical simplicity of the DNAm‐based method in addition to its nonlethality is advantageous to apply (Polanowski et al. 2014; Beal et al. 2019; García‐Vernet et al. 2021; Robeck et al. 2021). Furthermore, for species for which an age estimation model has already been constructed, the age structure of the populations with no chronological age data may be constructed.

**FIGURE 1:**
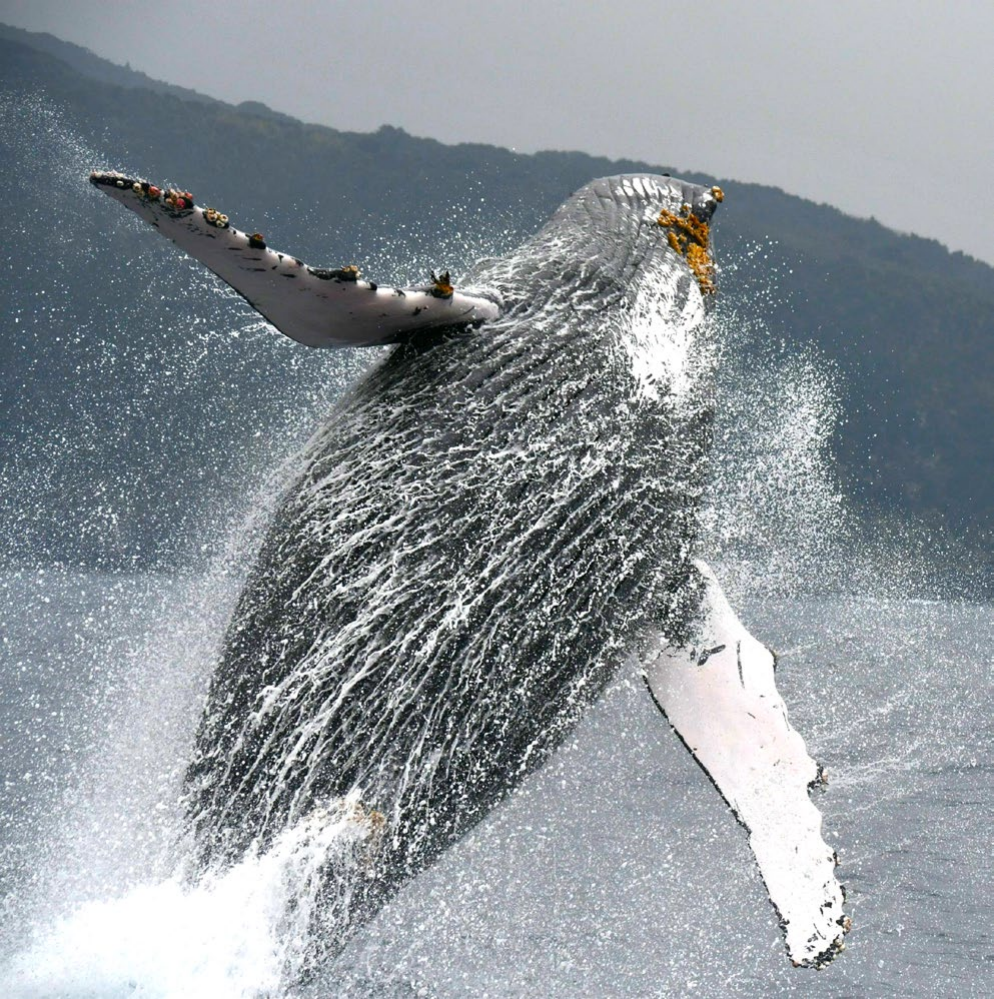
Humpback whale breaching off Hachijojima Island, Tokyo Metropolis, Japan. Photo credit: Hachijojima Island Tourism Association, Hachijo Town Office, and Tokyo University of Marine Science and Technology.

The humpback whale ( *Megaptera novaeangliae* , Figure 1) is a species of baleen whale and the only species in the genus *Megaptera*. They live in the major oceans around the world and undergo seasonal migrations to high‐latitude waters for feeding in the summer and low‐latitude waters for breeding in the winter (Dawbin 1966). At least four breeding areas are known in the North Pacific: Asia (western North Pacific), Hawaii (central North Pacific), Mexico, and Central America (eastern North Pacific) (Calambokidis et al. 2008). The breeding areas are characterized by shallow waters less than 200 m deep, the presence of adult male groups exhibiting aggressive behavior competing for mature females to mate, and the presence of mother and calf pairs (Dawbin 1966; Ersts and Rosenbaum 2003). Females give birth to calves after a gestation period of approximately 11.5 months and nurse their calves for approximately 1 year before weaning (Clapham and Mayo 1990; Clapham et al. 1999; Glockner‐Ferrari and Ferrari 1990). The age at sexual maturity was estimated to be approximately 5 years (Nishiwaki 1959; Clapham 1992), although some argued for older ages (Gabriele, Straley, and Neilson 2007; Best 2011). Although the lifespan of this species is not fully understood, the oldest reported age was 95 years old based on age estimation using earplugs (Chittleborough 1965). Humpback whales are the first cetaceans for whom a DNAm‐based age estimation method was applied, and an age estimation model (HEAA; Humpback Epigenetic Age Assay) has been developed as well and applied to the population off eastern Australia (Polanowski et al. 2014). The oldest individual off Raoul Island, New Zealand, was estimated to be 67 years old using HEAA (Riekkola et al. 2018).

In the winter of 2015, a number of migrating humpback whales suddenly emerged in the water of Hachijojima Island, Tokyo Metropolis, Japan. Before that, they had rarely been observed in the area, and, since then, a cumulative total of approximately 200–500 whales have been observed each year (Katsumata et al. 2021). Male competitive behavior for mating has been confirmed, suggesting their use of this water as a breeding area (Katsumata et al. 2021). If they are using this area as their breeding ground, this finding would be quite significant because it indicates that the breeding area of the western North Pacific population has been expanding northward beyond the traditionally known Okinawa Islands, Amami Oshima Island, and the Ogasawara Archipelago surrounding Japan (approximately 1400, 1100, and 700 km apart in straight lines, respectively, Kobayashi et al. 2022). Although the exact reason for the migration to Hachijojima Island is still unclear, one reason could be the observed recovery from the significant population decline due to commercial whaling in the 20th century. It is thought that the number of humpback whales in the North Pacific reached the carrying capacity in 2012 (Cheeseman et al. 2024). As a result, the traditional wintering grounds could reach the carrying capacity for suitable breeding areas, and some individuals may move to new areas to avoid area competition. Additionally, because the Kuroshio Large Meander has continued since its start in 2017 (Qiu, Chen, and Oka 2023), the Kuroshio Current (the subtropical western boundary current with warm, high‐salinity water) now often flows north of Hachijojima Island compared to the flow path of the past normal seasons. This change likely increased the water temperature around the island, which may create suitable environmental conditions for wintering humpback whales. The expansion of the biological knowledge of humpback whales that migrate to Hachijojima Island is urgent to ensure the effective management of this species in the future.

This study aimed to show an example of the practical use of the DNAm‐based age estimation method in ecological studies by conducting a preliminary analysis of the age structure of humpback whales migrating to Hachijojima Island. For populations like those that suddenly appeared on Hachijojima Island, determining their ages based on natural markings is basically impossible because of the lack of prior long‐term life records. This study is significant because it demonstrated that, despite such a situation, the DNAm‐based age estimation method is very effective in estimating age. In other words, this study successfully applied a practical, nonlethal age estimation method to a wild population whose age information is unavailable. This study also discussed whether the water around Hachijojima Island has the potential to function as a new wintering area for humpback whales.

## 2 Materials and Methods

### 2.1 Biopsy Samples

**FIGURE 2:**
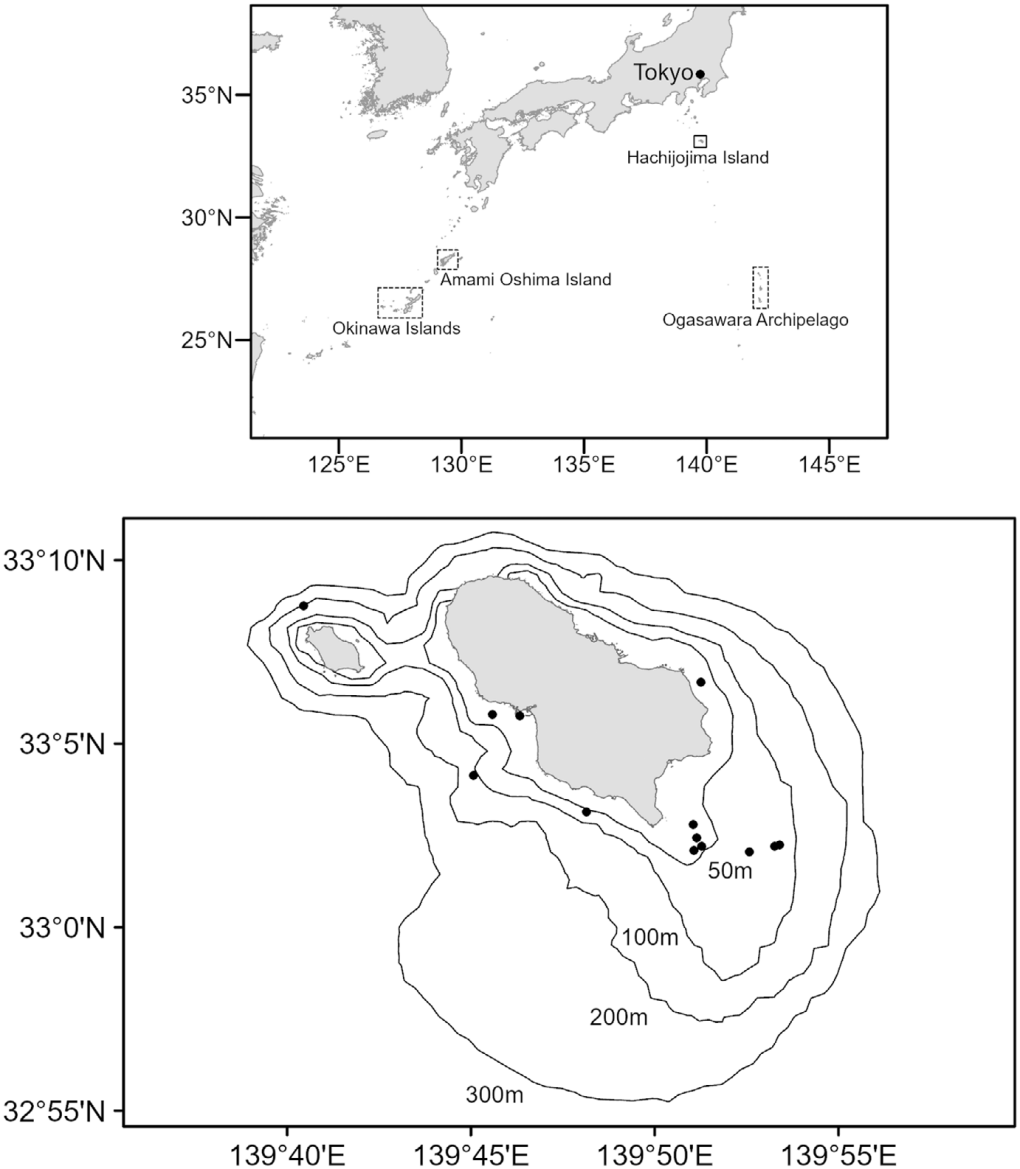
Wintering grounds of humpback whales around Japan (top) and the sighting positions of humpback whales in this study (black circles) and bottom depth contour lines (50, 100, 200, and 300 m; below).

Hachijojima Island is located approximately 300 km south of the center of Tokyo Metropolis (Figure 2). Since 2016, the year after the migration of many humpback whales was observed, we have been conducting whale research mainly through sighting surveys. Three researchers boarded a 15 m long, 12 GT vessel, and 3‐ to 4‐day long surveys were conducted at 2‐week intervals, twice a month from November to April. The survey area was mainly within two nautical miles (approximately 3.7 km) of the coast where humpback whales were concentrated. This study used 26 biopsy samples collected in the 2017/18, 2018/19, 2020/21, and 2021/22 seasons (Table 1). Regarding the timing, 80% of the samples were collected in the latter half of each season (February–April) primarily because the number of sightings was relatively low in the former half. Biopsy samples were collected using a crossbow (MK160‐B, Mang Kung, the Netherlands or TITAN M1, Tenpoint, United States) and a buoyant arrow fitted with a skin sampling tip. The firing range was within approximately 20 m. The skin tissue was collected from the back of the whale when the whale appeared above the water surface for breathing because DNAm frequencies obtained from the same CpG site of the same gene could differ between the skin samples collected from the different parts of the body, for example, ventral side and dorsal side (Goto, Kitakado, and Pastene 2020). The sample collection method followed the guidelines established by the Society for Marine Mammalogy (The Society for Marine Mammalogy 2023). After shooting the arrow at the whale, the arrow was collected using a dip net. If the sampling attempt succeeded, the tip of the arrow was placed in a plastic bag with a zipper, kept cool in a cooler box with an ice pack, and brought back to land. On land, the sample was placed in a vial and stored at −60°C or in a vial filled with 70% ethanol and stored at room temperature. Individual identification of the biopsy tissues was determined using 14 microsatellite DNA markers (Kanda et al., in prep.). Four cases (the sample IDs 2a and 2b, 7a and 7b, 16a and 16b, and 21a and 21b) were duplicate sampling from the same individual collected at different shooting opportunities because all 14 markers showed the same genotypes in each case, resulting in a total of 21 unique individuals. In addition to that, co‐amplification of a sex‐specific marker located on the Y chromosome and one of the 14 microsatellite DNA markers determined the sample's sex makeup (Kanda et al. in prep.), resulting in 15 males and 6 females. Furthermore, during the survey, the sample IDs 13 (calf) and 14 (mother) were sighted as a mother–calf pair, and the genetic data confirmed so because these two individuals shared at least one of the two alleles at all 14 microsatellite DNA markers (Kanda et al. in prep.).

**TABLE 1:** Biopsy samples of Humpback whales collected off Hachijojima Island.

Sample ID	Date sampled (y/m/d)	Sex^c^
1	2018/1/8	Male
2a^a^	2018/3/3	Female
2b^a^	2018/3/3	Female
3	2018/3/3	Female
4	2018/3/14	Male
5	2019/2/22	Male
6	2019/2/22	Female
7a^a^	2020/11/25	Male
7b^a^	2020/11/25	Male
8	2020/12/8	Female
9	2021/3/18	Male
10	2021/3/19	Male
11	2021/4/8	Male
12	2021/4/8	Male
13^b^	2021/4/9	Male
14^b^	2021/4/9	Female
15	2021/4/9	Male
16a^a^	2021/4/9	Male
16b^a^	2021/4/9	Male
17	2021/4/9	Male
18	2022/1/9	Female
19	2022/2/1	Male
20	2022/2/18	Male
21a^a^	2022/4/26	Male
21b^a^	2022/4/26	Male

^a^ Genetic analysis confirmed that 2a and 2b, 7a and 7b, 16a and 16b, and 21a and 21b were different biopsy samples, respectively, from the same individual collected at different shooting opportunities.

^b^ A sighting survey on board and genetic analysis afterward confirmed that 13 and 14 were a mother (14) and calf (13) pair.

^c^ Genetic analysis identified their sex.

### 2.2 Age Estimation Model

Polanowski et al. (2014) analyzed 45 humpback whales of known age and DNAm frequencies at three age‐related genes (*GRIA2*, *CDKN2A*, and *TET2*) in humans. They found that the CpG sites (*GRIA2* + 202, *CDKN2A* + 297, *TET2* + 31) located in the promoter regions of the three genes above had a similar age correlation in humpback whales and used these results to develop HEAA for humpback whales (Equation (1)):

(1) $$\left(\text{Estimated}\;\mathrm{age}\right)=5.4717\left(x\right)+3.9705\left(y\right)-0.6793\left(z\right)+1.4695$$

where *x*, *y*, and *z* refer to the DNAm frequencies (%) of *GRIA2* + 202, *CDKN2A* + 297, and *TET2* + 31, respectively. The estimation accuracy of HEAA was verified by the leave‐one‐out cross validation (LOOCV) method (Picard and Dennis Cook 1984): the mean error between known age and estimated age was 3.75 years, the standard deviation of the mean error between known age and estimated age was 2.99 years, and the 95% confidence interval for estimated age was 8.95 (Polanowski et al. 2014). In the present study, referring to Polanowski et al. (2014), we used HEAA with the DNAm frequencies in *GRIA2* + 202, *CDKN2A* + 297, and *TET2* + 31 to estimate the age of the Hachijojima humpback whales. We did not measure DNAm frequency using the same pyrosequencer as in Polanowski et al. (2014); instead, we used the next‐generation sequencer Miseq (Illumina, USA), which has a higher throughput.

### 2.3 Measurement of DNA Methylation Frequency

Approximately 30 mg of the biopsy skin sample was used to extract genomic DNA using the Puregene Tissue Kit (QIAGEN, Germany). We prepared nucleic acid fragments (libraries) for next‐generation sequencing analysis as follows. Firstly, we applied bisulfite treatment to genomic DNA using the Methyl Detector Bisulfite Modification Kit (Active Motif, USA). Before the bisulfite treatment, the genomic DNA was fragmented using a restriction enzyme (MseI) to increase the efficiency of the treatment. Secondly, we conducted the first PCR using the GoTaq DNA Polymerase system (Promega, USA) to amplify the target gene region and attach a gene‐specific adapter sequence (Table 2). The presence of the amplified PCR product was confirmed by 2% agarose gel electrophoresis, and the amplified DNA was cleaned with magnetic beads using AMPure XP (Beckman Coulter, USA). Thirdly, we conducted the second PCR (Table 2) using the GoTaq DNA Polymerase system (Promega, USA) with the first PCR product after magnetic bead purification as a template to attach an index sequence for sample identification (Illumina 2015) and an adapter sequence that allowed the library to bind on the flow cell of a next‐generation sequencer. The second PCR product was cleaned with magnetic beads using AMPure XP. These three processes created the gene‐specific library to analyze each of the three genes. After bead purification, the library was subjected to fragment analysis using the D1000 Screen Tape assay (Agilent, USA) to confirm the library size. After the adjustment of library concentration using the Qubit ds BR assay (Thermo Fisher Scientific, USA), sequencing was performed using MiSeq with Miseq Reagent Kits v2 (Illumina, USA). The read length was 250 bp × 2. We referred to coverage depth as the number of times the same nucleotide sequence was read, and we used it as an index of the detection precision of DNAm frequency. A deeper coverage depth, thus, provided more confidence in the level of DNAm frequency detected.

**TABLE 2:** Sequence of the PCR primers used for the first PCR and second PCR in this study.

Primer	Primer sequences
1st PCR	
*GRIA2*	
Forward: 5'‐ACACTCTTTCCCTACACGACGCTCTTCCGATCTGTGTGTGAGTGTATGGG‐3′	
Reverse: 5′‐GTGACTGGAGTTCAGACGTGTGCTCTTCCGATCTCCCTATTTCCCAAATCCTAC‐3′	
*CDKN2A*	
Forward: 5′‐ACACTCTTTCCCTACACGACGCTCTTCCGATCTAATGATTTTTGGTAAAGGGGAGAT‐3′	
Reverse: 5′‐GTGACTGGAGTTCAGACGTGTGCTCTTCCGATCTCCCCATATACTTTTCAATCCTCC‐3′	
*TET2*	
Forward: 5′‐ACACTCTTTCCCTACACGACGCTCTTCCGATCTGTGGTTAAAGTAAATAGAAGGT‐3′	
Reverse: 5′‐GTGACTGGAGTTCAGACGTGTGCTCTTCCGATCTCAAAAACACTCCCCAATTTC‐3′	
2nd PCR	
Forward: 5′‐AATGATACGGCGACCACCGAGATCTACAC‐index 2‐ACACTCTTTCCCTACACGACGC‐3′	
Reverse: 5′‐CAAGCAGAAGACGGCATACGAGAT‐index 1‐GTGACTGGAGTTCAGACGTGTG‐3′	

### 2.4 Data Processing

The raw data (fastq.gz file) output from the next‐generation sequencer was decompressed using the gunzip code. Cutadapt 1.18 (Martin 2011), implemented in Python, removed adapter sequences at both ends. Sickle 1.33 (Joshi and Fass 2011), a quality‐based trimming tool, removed reads with a read length of ≤ 40 bases as well as bases with a quality score of ≤ Q20. Paired‐end sequencing was used to analyze the library from both ends; thus, a single nucleic acid fragment had reads in two directions from both ends toward the inside. Flash2 2.2.00 (Magoc and Salzberg 2011) detected overlapping parts of the reads from both directions and merged the two reads into one, followed by the file conversion from fastq format to fasta format. Bowtie2 2.3.5.1 (Langmead and Salzberg 2012) created reference sequences for the three targeted genes (*GRIA2*, *CDKN2A*, and *TET2*) and mapped the fasta‐converted file to the reference sequences. Bismark 0.23.1 (Krueger and Andrews 2011) mapped the bisulfite treated sequences to the reference sequence and counted the number of methylated bases to determine the DNAm frequency. Finally, we substituted the DNAm frequency obtained in this manner into the abovementioned HEAA to estimate the age of the humpback whale.

## 3 Results and Discussion

The DNAm frequencies of each gene among the 26 biopsy samples were 0.66–2.66 (mean: 1.71) in *GRIA2*, 1.34–4.64 (mean: 2.22) in *CDKN2A*, and 5.59–19.19 (mean: 11.37) in *TET2* (Table 3). These values were low in *GRIA2* and *CDKN2A* but remained high in *TET2*. This observation was consistent with Polanowski et al. (2014) that analyzed DNA methylation for the same whale species and genes in this study.

**TABLE 3:** DNAm frequency and estimated chronological age of the humpback whale samples collected in Hachijojima Island. The age was estimated using the age estimation model, Humpback Epigenetic Age Assay (HEAA, Polanowski et al. 2014).

Sample ID	*GRIA2*		*CDKN2A*		*TET2*		Estimated age	95% CI
	DNAm frequency (%)	Coverage	DNAm frequency (%)	Coverage	DNAm frequency (%)	Coverage		
1	1.60	48,420	2.04	22,260	7.34	26,496	13.34	8.87–17.81
2a	1.59	24,334	2.03	22,716	11.80	16,883	10.21	5.74–14.68
2b	1.49	18,436	2.06	29,655	16.56	22,792	6.55	2.08–11.02
3	1.44	19,076	1.67	43,217	13.69	27,572	6.68	2.21–11.15
4	2.25	20,035	2.76	17,315	8.48	30,640	18.98	14.51–23.45
5	2.24	17,826	2.62	21,377	9.03	38,823	17.99	13.52–22.46
6	1.48	21,270	1.88	14,074	19.19	44,816	4.00	0.00–8.47
7a	1.50	13,543	1.76	24,649	10.33	24,956	9.65	5.18–14.12
7b	1.76	19,994	1.91	25,090	9.87	31,872	11.98	7.51–16.45
8	1.77	17,805	1.99	18,078	9.54	15,539	12.58	8.11–17.05
9	0.89	21,703	1.34	14,625	12.82	22,757	2.95	0.00–7.42
10	1.95	22,460	1.70	21,824	12.60	30,447	10.33	5.86–14.80
11	1.98	15,554	2.63	14,938	15.62	19,611	12.14	7.67–16.61
12	1.72	16,008	1.92	21,130	10.78	27,109	11.18	6.71–15.65
13	0.66	22,376	1.99	25,506	11.31	17,658	5.30	0.83–9.77
14	2.66	24,223	4.64	31,651	5.96	18,329	30.40	25.93–34.87
15	2.29	24,402	2.25	16,600	5.59	29,588	19.14	14.67–23.61
16a	2.61	24,452	2.57	28,260	15.34	10,249	15.53	11.06–20.00
16b	1.91	11,007	2.48	65,940	11.41	37,026	14.02	9.55–18.49
17	1.50	15,201	2.23	36,385	10.78	32,444	11.21	6.74–15.68
18	1.07	14,132	1.85	44,358	11.53	45,768	6.84	2.37–11.31
19	1.52	15,664	2.06	37,197	10.46	62,287	10.86	6.39–15.33
20	1.82	12,911	2.43	33,380	12.37	32,636	12.67	8.20–17.14
21a	1.96	29,249	2.28	29,356	11.53	50,290	13.41	8.94–17.88
21b	1.14	17,822	2.38	29,959	10.38	29,817	10.11	5.64–14.58

Efficient amplification using PCR and the use of next‐generation sequencing increased the coverage depth in this study. The coverage obtained to calculate the DNAm frequency in each sample was 11,007–48,420 (mean: 20,316) for *GRIA2*, 14,074–65,940 (mean: 27,582) for *CDKN2A*, and 10,249–62,287 (mean: 29,856) for *TET2* (Table 3). Coverages of over 10,000 were obtained from all the samples. Compared to the pyrosequencing used by Polanowski et al. (2014), the next‐generation sequencing was thought to be a better method because it offered higher throughput (Moser et al. 2020).

The reliability of the estimated ages from HEAA with the obtained DNAm frequencies was examined by comparing the ages of different biopsies that were collected from the same individual at different shooting opportunities. Sample combinations that fell under this condition were four individuals (sample IDs 2a and 2b, 7a and 7b, 16a and 16b, and 21a and 21b). The differences in estimated age between these samples were 1.51 years at a minimum and 3.66 years at a maximum, which was not considered a large difference given the respective 95% confidence intervals (Table 3). Therefore, the estimated ages were thought reliable in this study. The mean between two different biopsy samples obtained from the same individual was used as the estimated age of each of the individuals: 2a/2b being 8.38 years old, 7a/7b being 10.82 years old, 16a/16b being 14.78 years old, and 21a/21b being 11.76 years old. The validity of the estimated age was then judged using a mother–calf pair sample. Sample ID 13 and 14 had been confirmed as a pair of mother (14) and calf (13) through a sighting survey and subsequent genetic analysis. Their estimated ages were 30.40 and 5.30, respectively (Table 3). The estimated age of another sample that was thought to be a calf during the sighting survey (sample ID 9) was 2.95 (Table 3). Since humpback whales become independent from their mothers approximately 1 year after birth, the actual age of the calf swimming with its mother should have been ≤ 1 year. The estimated ages (2.95 and 5.30) were within the 95% confidence interval. We, therefore, thought that the age difference between parents and calves was adequately described. Based on these reliability and validity results, we inferred that our DNAm‐based age estimation reasonably reflected the chronological age of the Hachijojima humpback whales.

**FIGURE 3:**
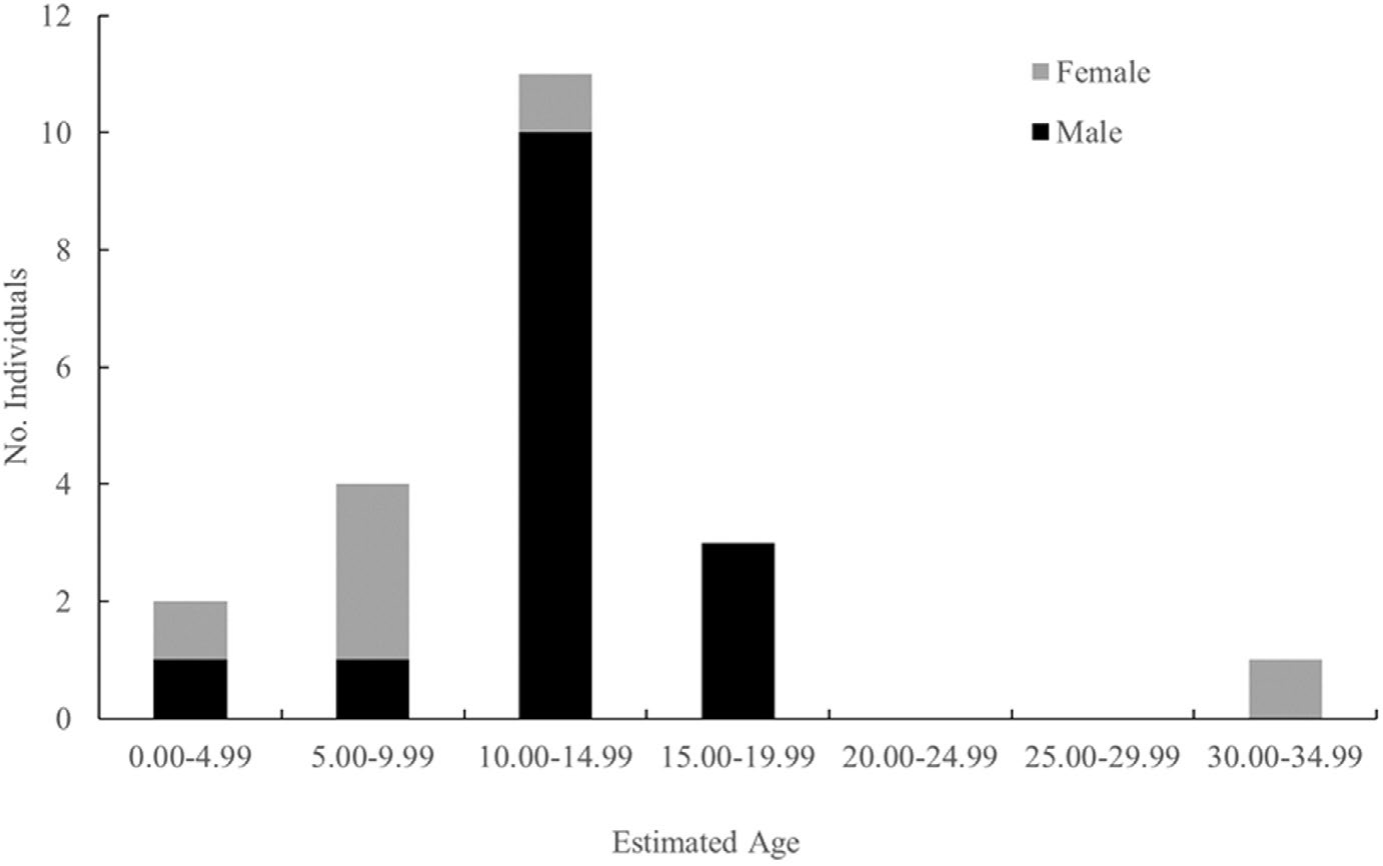
Age structure of humpback whale samples biopsied around Hachijojima Island. The age was estimated using the Humpback Epigenetic Age Assay (HEAA, Polanowski et al. 2014).

The age range of the 21 humpback whales used in this study was 2.95–30.40, with a mean age of 12.02. Of these, the estimated age of the 15 males was 2.95–19.14 years (mean: 12.23 years), and the estimated age of the six females was 4.00–30.40 years (mean: 11.48 years). We depicted the age distribution in 5‐year increments (Figure 3). Although no report on the age of sexual maturity in the western North Pacific population has been found in recent years, this depiction relied on Nishiwaki (1959) that stated that the age of sexual maturity for the population was approximately 5 years. Some indicated older age at sexually mature (see Best 2011), and the age at sexual maturity could vary due to complex factors including the nutritional status of the population. Nevertheless, the 5‐year increment was thought to be acceptable to separate sexually mature and immature individuals for convenience. The result showed that the age structure of all 21 individuals was close to a normal distribution, with a peak at the 10.00–14.99 years age class (Figure 3), indicating the dominance of young sexually mature males among the samples.

Given the lifespan of humpback whales, a few older individuals were observed. Unless there is a possibility of no migration of older individuals in this location, the high proportion of young, sexually mature males may be a feature of humpback whales migrating to Hachijojima Island. Two other possibilities for a few older individuals can be raised. First, there may be the geographic and timing influence on the sampling. For safety reasons, biopsy sampling was often conducted in the southern area of Hachijojima Island because the sea conditions of the southern area were better than the northern area due to strong northerly winds during the winter season. The southern area of the island has a wider area of water shallower than 200 m compared to the northern side, and thus mother–calf groups, as well as relatively young individuals, could occupy there considering their ecology observed in other regions (Craig and Herman 2000; Ersts and Rosenbaum 2003). Regarding the timing of sample collection, humpback whales are also known to visit breeding areas at different times depending on their age, sex, and maturity (Craig et al. 2003). Second, an inherent bias might still exist in HEAA itself (Polanowski et al. 2014) because the proportion of older individuals in the calibration samples was low when developing HEAA. HEAA may underestimate the ages of older age individuals.

The age structure can be used as an index of whether the status of the population is healthy or not (Polanowski et al. 2014; Rughetti 2016; Jones et al. 2018). Humpback whales were hunted in major oceans worldwide, including around Japan, until commercial hunting was banned by the International Whaling Commission in 1966, and the number of humpback whales decreased due to overfishing (Johnson and Wolman 1984). Commercial whalers, in general, selectively hunt for larger individuals, causing the peak of age structure to shift to younger age classes over time (Clements et al. 2017). Because the DNAm‐based age estimation itself involves some inherent errors between the estimated and actual age, the construction of age structure for a population by a certain increment, as described in this study, could be more useful than trying to accurately estimate the age of each individual to understand the characteristics of the whales in the area. Further studies with increasing sample sizes are awaited to compare current and past age distributions.

Aggressive behavior for mating and whale songs have been observed in humpback whales migrating to Hachijojima Island (Katsumata et al. 2021). Likewise, mother–calf pairs have been confirmed as described in this study. There are shallow waters with a depth of less than 200 m, particularly in the southern area of the island. These are features of wintering areas for humpback whales (Craig and Herman 2000; Ersts and Rosenbaum 2003). Therefore, the water off Hachijojima Island is highly likely being used as a new wintering area, suggesting that the northern limit of the breeding range of humpback whales distributed around Japan is expanding. Herman (2017) stated that the wintering areas of humpback whales are generally stable worldwide, but this species has been observed in new areas. The change in the marine environment due to the Kuroshio Large Meander (Qiu, Chen, and Oka 2023) and the recent increase of humpback whales in the North Pacific (Cheeseman et al. 2024) could be contributing factors. Investigating the differences in the sex and age structure of individuals between new and existing areas is of biological and ecological interest. Further research, therefore, should compare these features between humpback whales off Hachijojima Island and those in other regions of the North Pacific including the Okinawa Islands, Amami Oshima Island, and the Ogasawara Archipelago.

The combination of nonlethal biopsy sampling and DNAm analysis allows the acquisition of age information relatively easily, even for species that no hunting is allowed or are endangered. Concerning the future management of humpback whales migrating to the water off Hachijojima Island, various kinds of data, such as sightings, photo IDs, biopsies, and acoustics have been accumulating since 2016. Combined use of these data with the results of this study should allow us to conduct comprehensive studies to better understand the humpback whales migrating to Hachijojima Island.

## Author Contributions

**Kohei Igarashi:** conceptualization (equal), data curation (equal), formal analysis (equal), investigation (equal), methodology (equal), validation (equal), visualization (equal), writing – original draft (equal), writing – review and editing (equal). **Atsushi Tanabe:** data curation (equal), formal analysis (equal), investigation (equal), methodology (equal), validation (equal). **Hiroeki Sahara:** conceptualization (equal), resources (equal), supervision (equal), writing – review and editing (equal). **Reiko Nozaki:** data curation (equal), formal analysis (equal), investigation (equal), methodology (equal). **Hidehiro Kondo:** formal analysis (equal), methodology (equal), supervision (equal), writing – review and editing (equal). **Taiki Katsumata:** data curation (equal), funding acquisition (equal), resources (equal), writing – original draft (equal). **Shingo Tamura:** funding acquisition (equal), project administration (equal), resources (equal), writing – review and editing (equal). **Tadashi Yamakoshi:** conceptualization (equal), funding acquisition (equal), project administration (equal), resources (equal), supervision (equal), writing – review and editing (equal). **Mizuki Mori:** data curation (equal), formal analysis (equal), investigation (equal), methodology (equal). **Marin Miyagi:** data curation (equal), formal analysis (equal), investigation (equal), methodology (equal). **Gen Nakamura:** funding acquisition (equal), supervision (equal), writing – review and editing (equal). **Naohisa Kanda:** conceptualization (equal), investigation (equal), methodology (equal), supervision (equal), validation (equal), writing – original draft (equal), writing – review and editing (equal). **Hiroto Murase:** conceptualization (equal), data curation (equal), formal analysis (equal), investigation (equal), project administration (equal), resources (equal), supervision (equal), visualization (equal), writing – original draft (equal), writing – review and editing (equal).

## Conflicts of Interest

The authors declare no conflicts of interest.

## Data Availability

Data that support the findings of this study are shown in Tables 1, 2, 3.
